# Influence of head models on neuromagnetic fields and inverse source localizations

**DOI:** 10.1186/1475-925X-5-55

**Published:** 2006-10-23

**Authors:** Ceon Ramon, Jens Haueisen, Paul H Schimpf

**Affiliations:** 1 Department of Electrical Engineering, University of Washington, Seattle, WA 98195, USA; 2 Institute of Biomedical Engineering and Informatics, Technical University Ilmenau, Germany; 3 School of Electrical Engineering and Computer Science, Washington State University, Spokane, WA 99202, USA

## Abstract

**Background:**

The magnetoencephalograms (MEGs) are mainly due to the source currents. However, there is a significant contribution to MEGs from the volume currents. The structure of the anatomical surfaces, e.g., gray and white matter, could severely influence the flow of volume currents in a head model. This, in turn, will also influence the MEGs and the inverse source localizations. This was examined in detail with three different human head models.

**Methods:**

Three finite element head models constructed from segmented MR images of an adult male subject were used for this study. These models were: (1) Model 1: full model with eleven tissues that included detailed structure of the scalp, hard and soft skull bone, CSF, gray and white matter and other prominent tissues, (2) the Model 2 was derived from the Model 1 in which the conductivity of gray matter was set equal to the white matter, i.e., a ten tissuetype model, (3) the Model 3 consisted of scalp, hard skull bone, CSF, gray and white matter, i.e., a five tissue-type model. The lead fields and MEGs due to dipolar sources in the motor cortex were computed for all three models. The dipolar sources were oriented normal to the cortical surface and had a dipole moment of 100 *μA meter*. The inverse source localizations were performed with an exhaustive search pattern in the motor cortex area. A set of 100 trial inverse runs was made covering the 3 cm cube motor cortex area in a random fashion. The Model 1 was used as a reference model.

**Results:**

The reference model (Model 1), as expected, performed best in localizing the sources in the motor cortex area. The Model 3 performed the worst. The mean source localization errors (MLEs) of the Model 3 were larger than the Model 1 or 2. The contour plots of the magnetic fields on top of the head were also different for all three models. The magnetic fields due to source currents were larger in magnitude as compared to the magnetic fields of volume currents.

**Discussion:**

These results indicate that the complexity of head models strongly influences the MEGs and the inverse source localizations. A more complex head model performs better in inverse source localizations as compared to a model with lesser tissue surfaces.

## Background

In a recent paper [1] we have shown that complexity of human head models significantly influence the scalp potential and the EEG inverse source localizations. In this companion work, we examine the influence of head models on neuromagnetic fields and on MEG inverse solutions. Similar to the previous results on EEG, the MEGs and the source localizations for MEGs are also influenced by the complexity of the head models. In the previous work the dipoles were oriented along the x, y and z directions. However, in the present work, the dipoles are oriented normal to the cortical surface which is a more realistic emulation of the cortical electrical activity. Here, we have also examined the contributions of the source currents and the volume currents on the MEG simulations and the inverse source localizations.

Earlier [1]we have reviewed the literature on influence of head models on EEG and MEG source localizations and lead field computations [2-6]. That literature review is also applicable here and a brief summary is given. In the past, a 3-compartment boundary element model of the head, or a 3-shell spherical model of the head or a five tissue-type finite element model of the head has been used for EEG and MEG studies. In general, previous studies have found that a more complex head model performs better than a less complex model in EEG and MEG simulations and in inverse source localization. These previous studies [5-7] show that more complex head models account for volume currents more precisely as compared to simpler, e.g., spherical, head models. Recently, a generalized head model based on symmetric BEM formulation, has also been proposed for EEG and MEG simulations [8]. It performs better than a nested volume BEM head model. However, it also lacks the capacity to accurately represent the cortical structures, such as, sulci and gyri in the brain.

The finite element method (FEM) head models are still preferable to accurately model the cortical structure and other tissue surfaces in the head as compared to BEM models. Thus, in summary, FEM models of the head are better suited to perform the proposed work. A five tissue-type FEM model of the head has been used earlier for efficient computations of the lead fields [4,5] and also for analyzing the effects of tissue conductivities on MEG forward and inverse simulations [6]. In comparison, our head models consist of eleven different tissues and have a potential to provide a more accurate simulations of EEGs and MEGs. These models are used in the study reported here.

In this work we are reporting our results on forward simulations of the radial and Cartesian *x, y *and *z *components of the magnetic fields. Inverse source localizations were also performed with these magnetic fields. The radial component of the magnetic field is the one which is usually measured with a whole-head SQUID biomagnetometer system. However, it is also becoming common to measure local *x, y, z *components of the field with vector biomagnetometers. This prompted us to include the simulation analysis of Cartesian components also which is very similar to our previous work on EEG simulations [1]. The coordinate orientations are: the *x *coordinate increases from anterior (front) to posterior (back), the *y *coordinate increases from superior (top) to inferior (bottom), the *z *coordinate increases from left to right. The origin is at the first (left) slice at the anterior and superior corner. Here radial refers to the magnetic field components which emanate out, approximately, radially from the scalp surface. It does not refer to the radial dipole orientations in the cortex.

## Methods

### Model constructions

Our model building details have been described earlier [1]. For the sake of completeness, these details are included here. Finite element models of the head were constructed from the segmented MRI (magnetic resonance imaging) slices of an adult male subject. The T1 weighted sagittal MRI slices with 3.2 mm thickness were collected with a 1.5 Tesla GE Signa scanner. The original MR slices were of 256 × 256 resolution with 1.0 mm size pixels [1,9]. A total of 51 contiguous slices was used. Eleven major tissues were identified in the image slices. The MR images were segmented by use of a semiautomatic tissue classification software developed by us [10]. After segmentation, the slices were checked by a radiologist for any errors and the segmentation was corrected as needed. Three- dimensional FEM models of the head were developed from these segmented images. For simulation studies, three FEM models were used:

Model 1: Full model with eleven tissuetypes,

Model 2: Full model with the conductivity of gray matter equal to white matter, i.e., a ten tissuetype model,

Model 3: Five tissue model consisting of scalp, hard skull, CSF, gray and white matter.

The motivation for the selection of these three models are that the conductivity of gray matter significantly influences the MEGs [7] and a five tissue model has become increasingly popular in forward and inverse computations [4,5,7]. The eleven tissues used in the Model 1 are: scalp, fat, muscle, hard skull bone, soft skull bone, gray matter, white matter, eyes, cerebellum, cerebrospinal fluid (CSF) and soft tissue.

**Figure 1:**
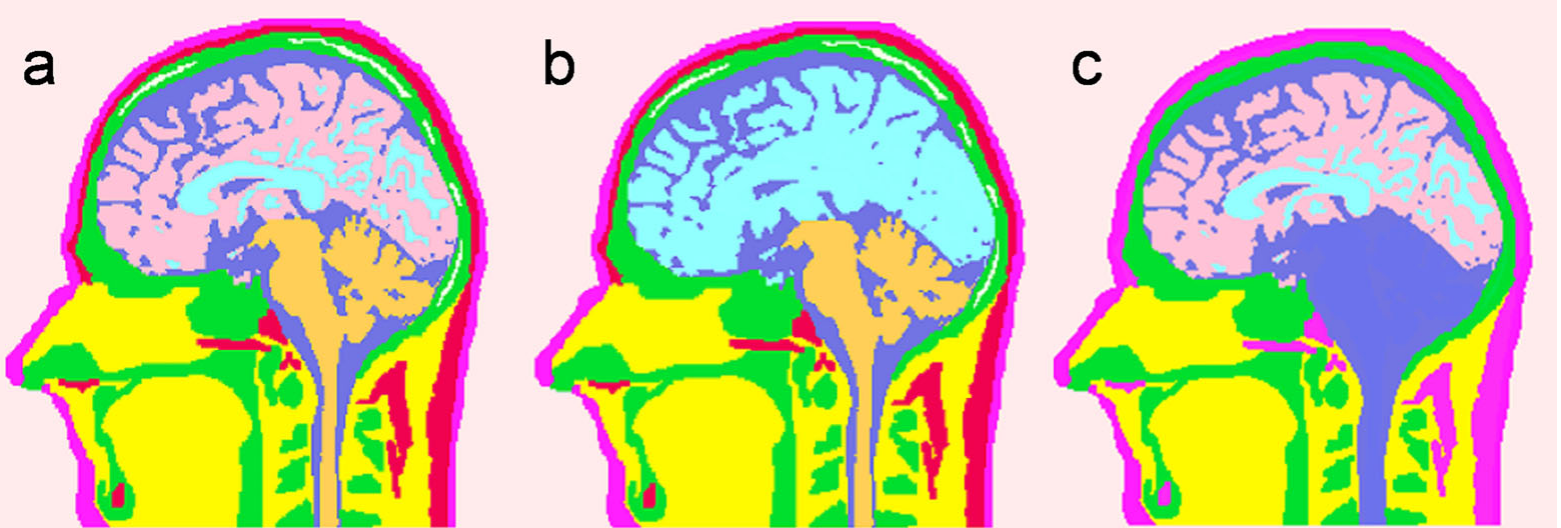
Human head models with varying tissue complexities. (a) Model 1 with eleven tissuetypes, (b) Model 2 with distinction between gray and white matter removed, (c) Model 3 with five major tissue-types.

Refer to Figure 1 for details of these three models. Figure 1a is for the Model 1. It has all the tissue surfaces intact. In Figure 1b for the Model 2, distinction between the gray and white matter boundaries has been eliminated. The Model 3 is shown in Figure 1c. It has no soft skull bone, cerebellum, muscle and the fat layer.

The Model 3 is composed of fewer tissue-types as compared with other models. The major tissues in this model are: scalp, hard skull, CSF, gray and white matter. This model was developed by replacing the other tissues in each slice with the nearby tissues. As an example, soft bone was replaced with the hard bone in the skull; cerebellum was replaced with CSF; fat layer near to the scalp was replaced with the scalp and eye sockets were replaced with soft tissue. Similarly, all tissues below the jaw in the Model 3 were treated as soft tissue while building the FEM model of the head. The Model 3 is similar to van Uitert's model [4]. Our choice to replace cerebellum with CSF in the Model 3 was based on a suggestion that if the tissue was damaged due to stroke, it will be eventually filled by the CSF. Thus, the model developed here will become a good reference model for stroke studies.

The segmented images were subsampled to a 2 × 2 mm resolution for building finite element models of the head. The finite element mesh for all three models was generated through the connection of all slices. All three models had a voxel resolution of 2 × 2 × 3.2 mm. The voxels were hexahedral, i.e., brick-shaped elements with linear basis functions. There were 835,584 hexahedral voxels and 865,332 nodes in each model. The tissue resistivity values used in the models are given in Table 1. These values have been used by us before in our head modeling studies [1,9] and are compiled from published values [11-13].

**Table 1:** Head tissue resistivity and conductivity values compiled from the literature [11–13].

**Tissue**	**Resistivity(*Ohm cm*)**	**Conductivity(*Siemens/cm*)**
Brain White Matter	700	1.428E-3
Brain Gray Matter	300	3.334E-3
Spinal Cord and Cerebellum	624	1.6026E-3
Cerebrospinal Fluid (CSF)	65	15.38E-3
Hard Bone	16000	6.25E-5
Soft Bone	2180	4.587E-4
Muscle	900	1.1112E-3
Fat	2500	4.0E-4
Eye	198	5.0505E-4
Scalp and Skin	230	4.3478E-3
Soft Tissue	576	1.7361E-3

Based on the grid in each model, a linear system of equations was set up and solved iteratively using an uniform finite element solver [14,15]. The current densities in each voxel of the head model were computed. A preconditioned conjugate gradient method was used for solving the linear system of equations. The convergence of the conjugate gradient solver was ensured by two criteria: first, the L2 norm of the system matrix of the linear system of equations had to drop so that the first five significant digits did not change anymore, and secondly, the potential difference had to decrease continuously during the iteration process.

### Lead field computations

The motor cortex within a volume of 3 cm cube was represented by 716 hexahedral voxels. The volume current densities in the whole model were computed for all dipole locations in the motor cortex. This was done by placing one dipole at a time at a node of the voxel and the volume current densities were computed. The dipole was oriented normal to the local cortical surface at that particular node. The dipole moment was 100 *μA-meter*. These dipoles were represented with an approximate Laplace formulation described elsewhere [16]. Using Biot-Savart law and the volume current densities, the magnetic fields were computed at the coil positions. Similarly, magnetic fields at the coil positions due to the dipolar source currents were also computed.

The lead fields at 145 MEG coil positions were computed for all three models due to dipolar sources in the motor cortex area. The MEG sensing coils were assumed to be radially 1.0 cm above the scalp. The MEG coil positions were above the EEG electrode positions on the scalp. Details of EEG electrode positions are described in our earlier paper [1]. These were generated by starting with the 82 sampling points of an extended EEG 10–20 layout, and visually interpolating an additional 63 points. These sensing coil positions were approximately uniformly distributed covering the whole head.

### Inverse source localizations

For inverse source localizations, first the simulated MEGs were generated for a given dipolar source. The model 1 was used as a reference model. The magnetic field due to the source current and the lead fields computed from the volume currents were added together. For each trial, a dipolar source with a random magnitude was placed at a given position in the motor cortex. The MEGs were simulated at 145 coil positions by multiplying the combined magnetic field of that particular dipole with its random magnitude. The uncorrelated Gaussian noise was added to achieve the desired signal to noise (SNR) ratio of the simulated MEGs. The SNR was defined as [1,16]:

SNR=10log⁡{var⁡(Vexact)σ2}     (1)
 MathType@MTEF@5@5@+=feaafiart1ev1aaatCvAUfKttLearuWrP9MDH5MBPbIqV92AaeXatLxBI9gBaebbnrfifHhDYfgasaacH8akY=wiFfYdH8Gipec8Eeeu0xXdbba9frFj0=OqFfea0dXdd9vqai=hGuQ8kuc9pgc9s8qqaq=dirpe0xb9q8qiLsFr0=vr0=vr0dc8meaabaqaciaacaGaaeqabaqabeGadaaakeaacqWGtbWucqWGobGtcqWGsbGucqGH9aqpcqaIXaqmcqaIWaamcyGGSbaBcqGGVbWBcqGGNbWzdaGadeqaamaalaaabaGagiODayNaeiyyaeMaeiOCaiNaeiikaGIaemOvay1aaSbaaSqaaiabdwgaLjabdIha4jabdggaHjabdogaJjabdsha0bqabaGccqGGPaqkaeaaiiGacqWFdpWCdaahaaWcbeqaaiabikdaYaaaaaaakiaawUhacaGL9baacaWLjaGaaCzcamaabmaabaacbeGae4xmaedacaGLOaGaayzkaaaaaa@4E41@

where *var*(*V*_*exact*_) is the variance of the simulated noisefree observations, and *σ*^2 ^is the variance of the added noise. Due to the addition of the noise, the simulated MEGs for the reference Model were very different from the lead fields as well as from the magnetic field of the source current.

These forward simulated MEGs were then used for inverse source localizations using the lead fields of three different models. For inversion, the magnetic field due to the source current and the lead fields computed from the volume currents were added together. The Model 1, as stated earlier, was used as a reference model. Inversions were performed with the least-squares technique. An exhaustive search pattern was used, i.e., inversion was performed for each possible source location in the motor cortex and the site producing the smallest residual norm was selected as the best possible source location. The inversions were performed with the *x, y, z *components and with the radial component of the magnetic field.

A set of 100 trial inverse runs was made covering a 3 cm cubic volume in the motor cortex in a random fashion. Each trail had a different intensity and a different location in that 3 cm cubic volume. All computations were performed on an Intel 3.2 GHz workstation with 1.2 gigabytes memory. Each run for the lead field computation took between 2–3 seconds. Postprocessing and visualizations were done using the Matlab software, version 7.1 (Mathworks, Inc., Natick, MA).

## Results

### Forward MEG simulations

**Figure 2:**
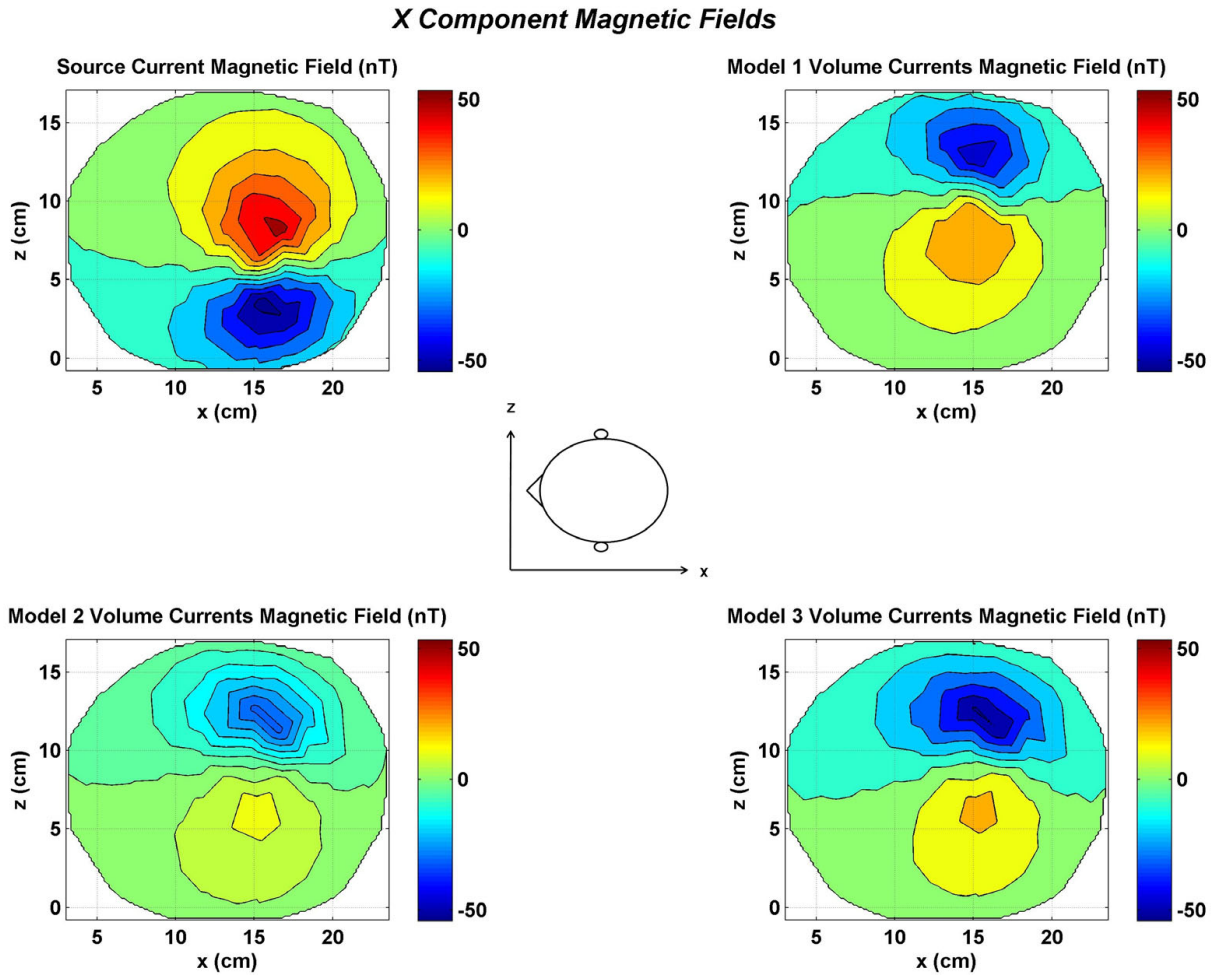
Contour plots of the *x *component of the magnetic fields at top of the head. All values are in nano Tesla *(nT)*. All plots have the same magnitude scale shown by color bars. (Top left) source current magnetic fields; (top right) magnetic field due to volume currents in the Model 1; (bottom left) magnetic field due to volume currents in the Model 2; (bottom right) magnetic field due to volume currents in the Model 3. The location of the positive and negative contour peaks for the source current and the volume currents are reversed.

**Figure 3:**
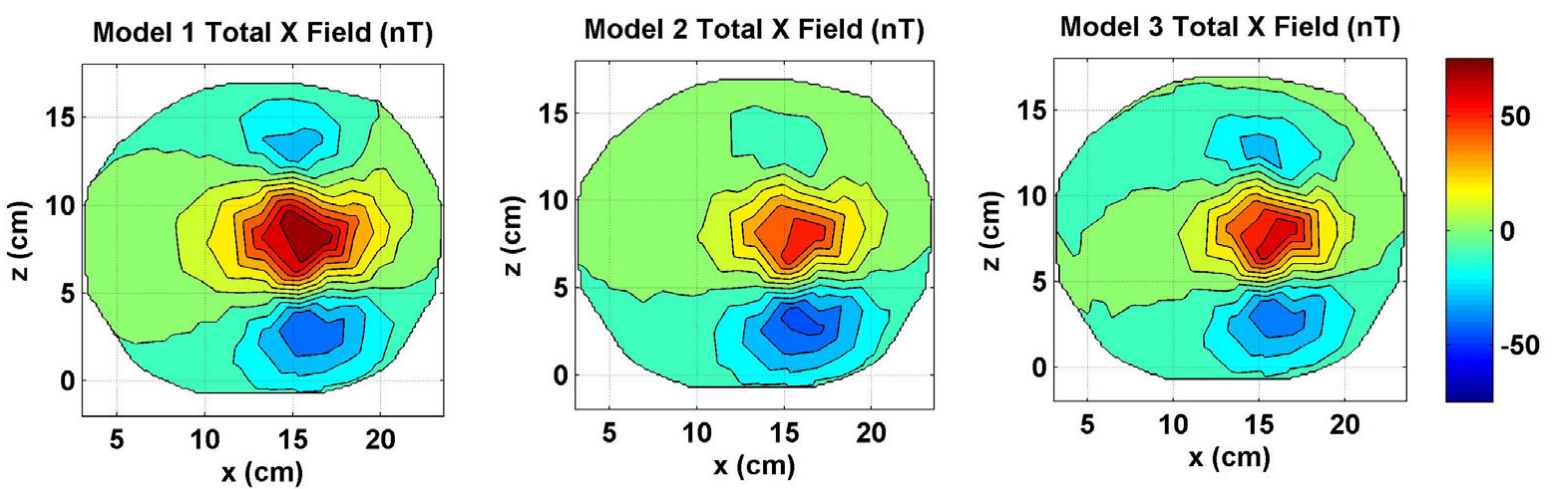
Combined *x *component of the magnetic field of source and volume currents for three models. Notice that the magnetic field due to source currents still dominates the contour plots. There are significant noticeable differences between the contour plots of models 1, 2 and 3. These differences are due to magnetic fields of volume currents. All values are in nano Tesla (*nT*).

**Figure 4:**
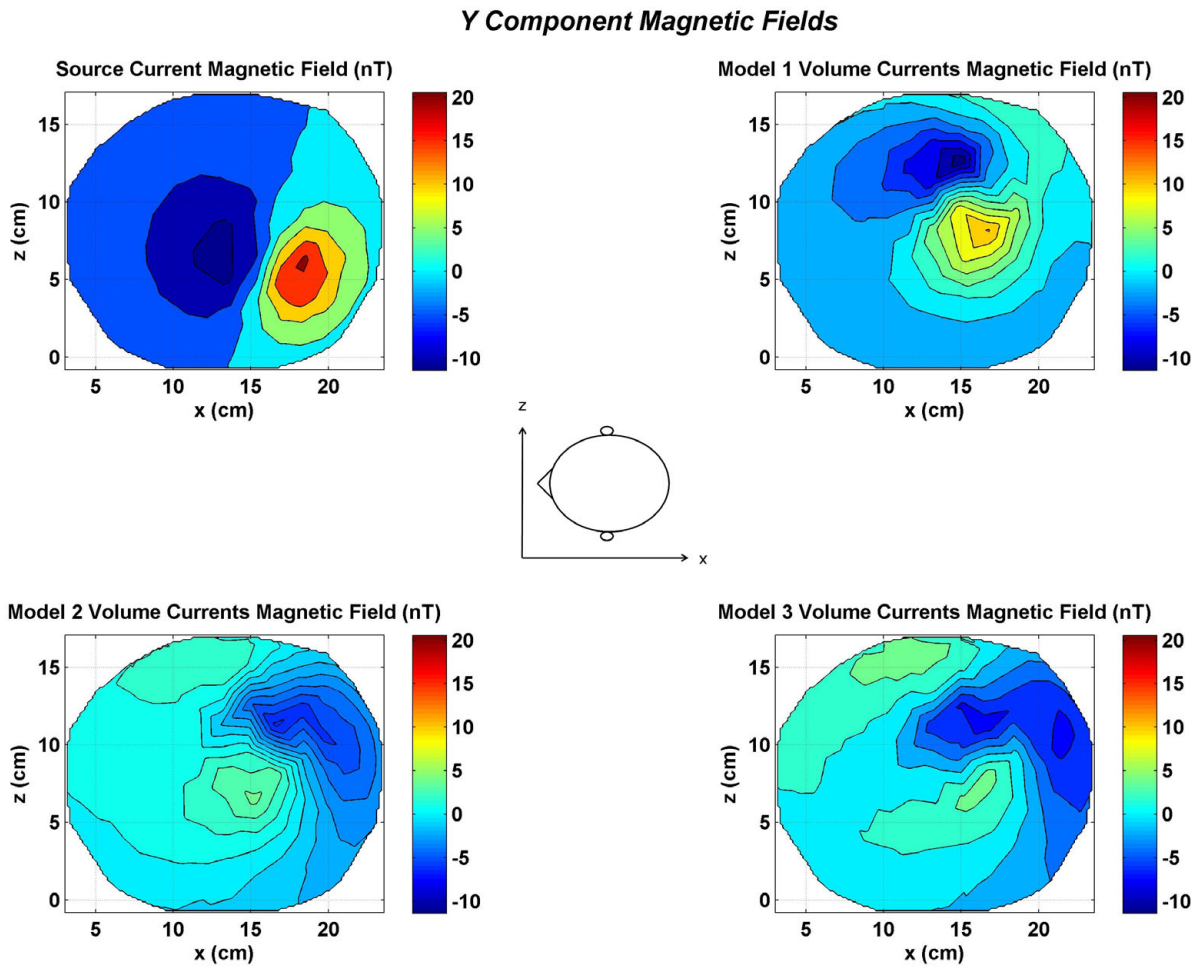
Contour plots of the *y *component of the magnetic fields at top of the head. All values are in nano Tesla (*nT*). All plots have the same magnitude scale shown by color bars. (Top left) source current magnetic fields; (top right) magnetic field due to volume currents in the Model 1; (bottom left) magnetic field due to volume currents in the Model 2; (bottom right) magnetic field due to volume currents in the Model 3.

**Figure 5:**
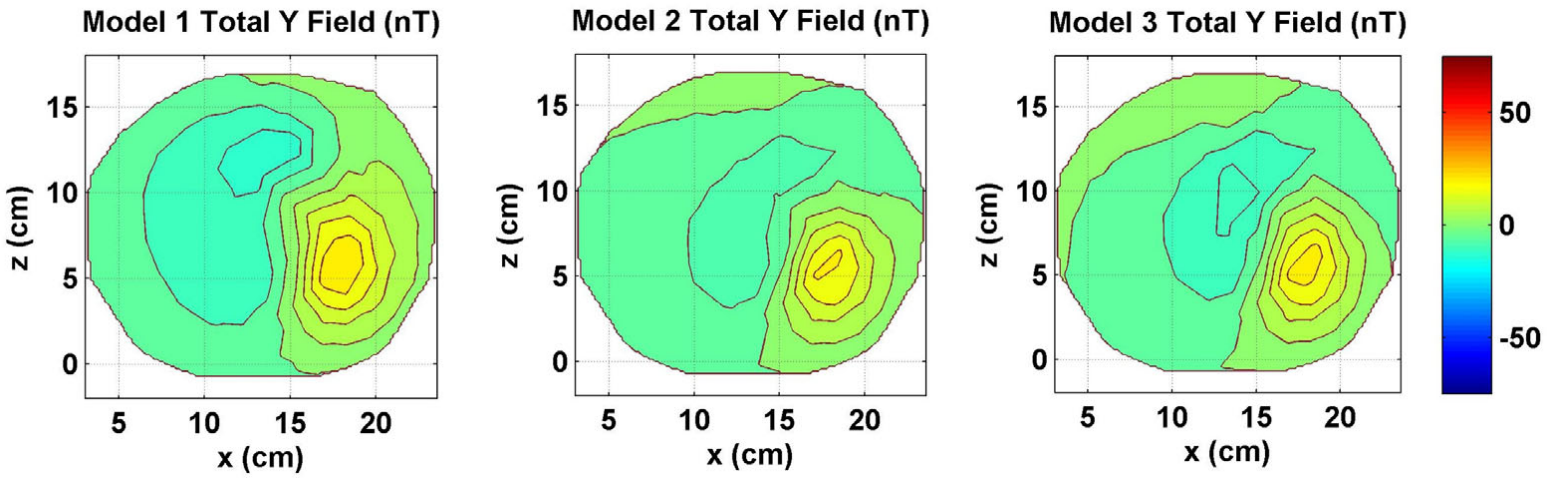
Combined *y *component of the magnetic field of source and volume currents for three models. All values are in nano Tesla (*nT*).

**Figure 6:**
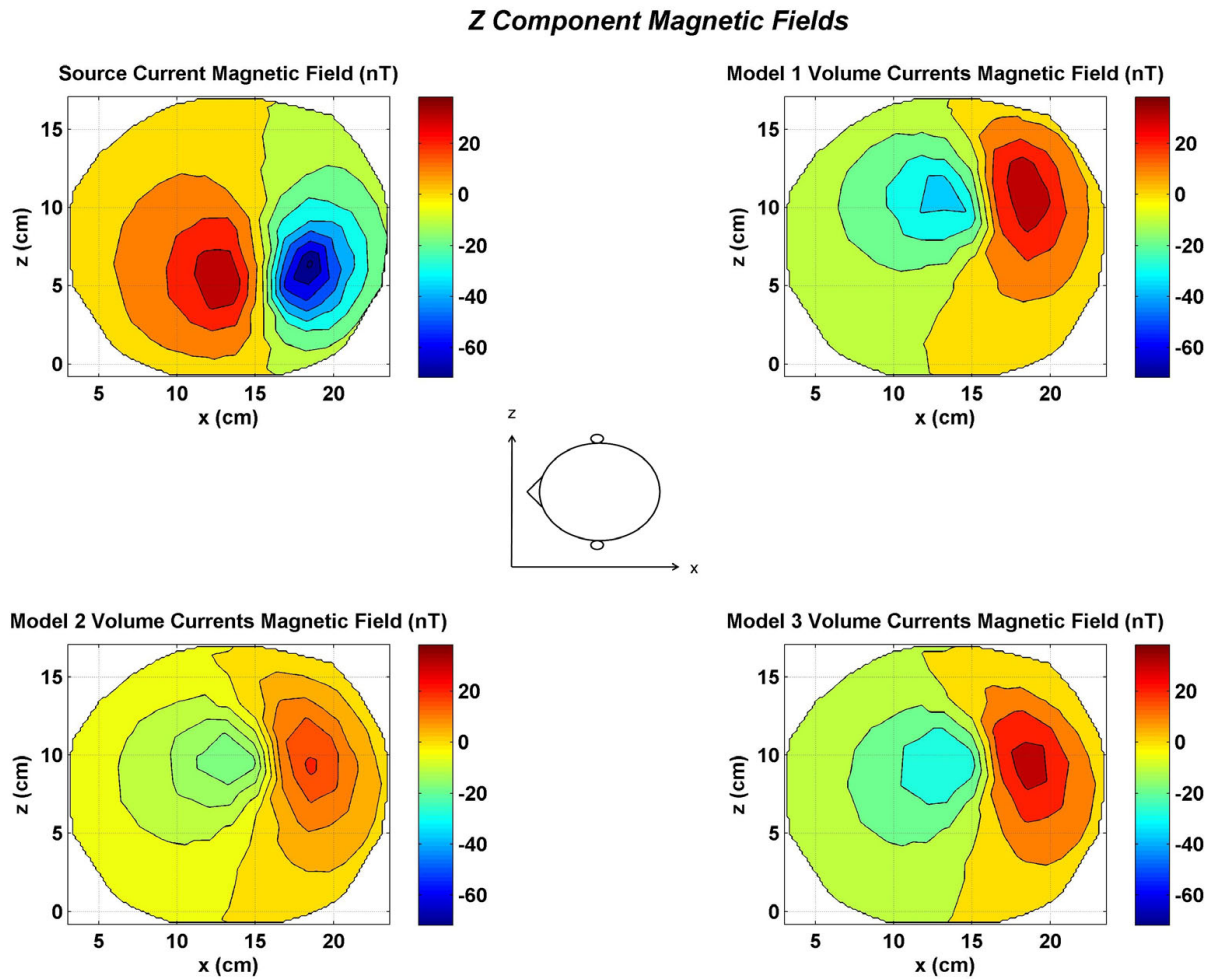
Contour plots of the *z *component of the magnetic fields at top of the head. All values are in nano Tesla *(nT)*. All plots have the same magnitude scale shown by color bars. (Top left) source current magnetic fields; (top right) magnetic field due to volume currents in the Model 1; (bottom left) magnetic field due to volume currents in the Model 2; (bottom right) magnetic field due to volume currents in the Model 3.

**Figure 7:**
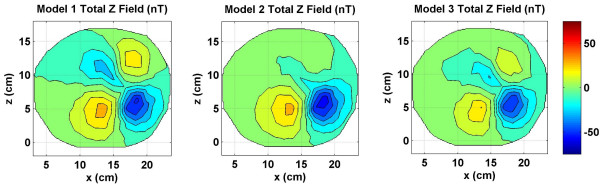
Combined *z *component of the magnetic field of source and volume currents for three models. All values are in nano Tesla (*nT*). The Model 1 has more features as compared to the other two models. It is due to the separate spatial locations of the peaks of the source and volume currents magnetic fields.

**Figure 8:**
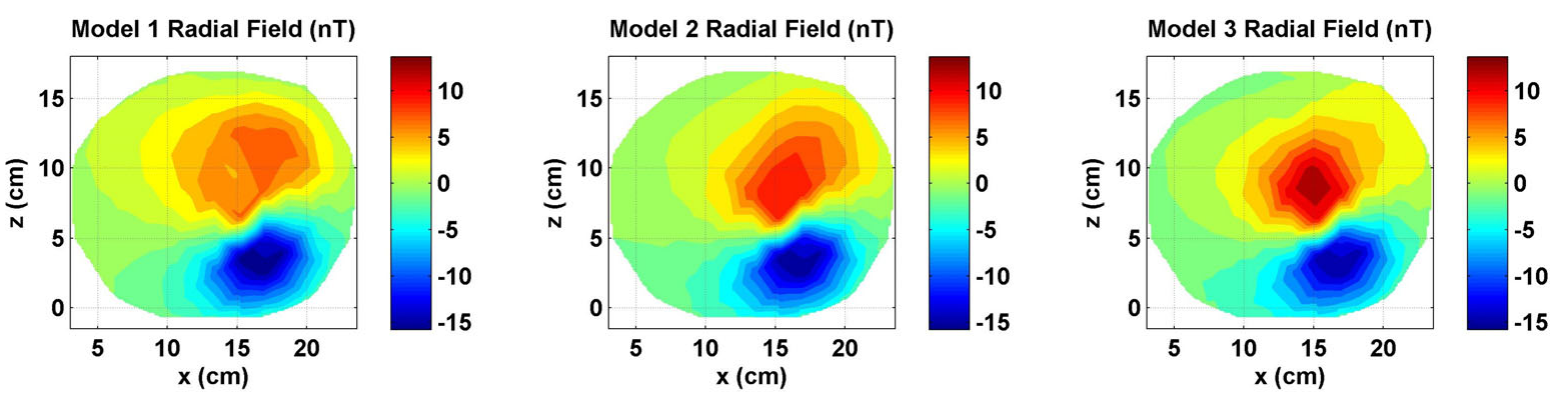
Radial component of the magnetic fields of all three models as one would measure with a multi-channel SQUID biomagnetometer. There are subtle noticeable differences between all three plots.

**Figure 9:**
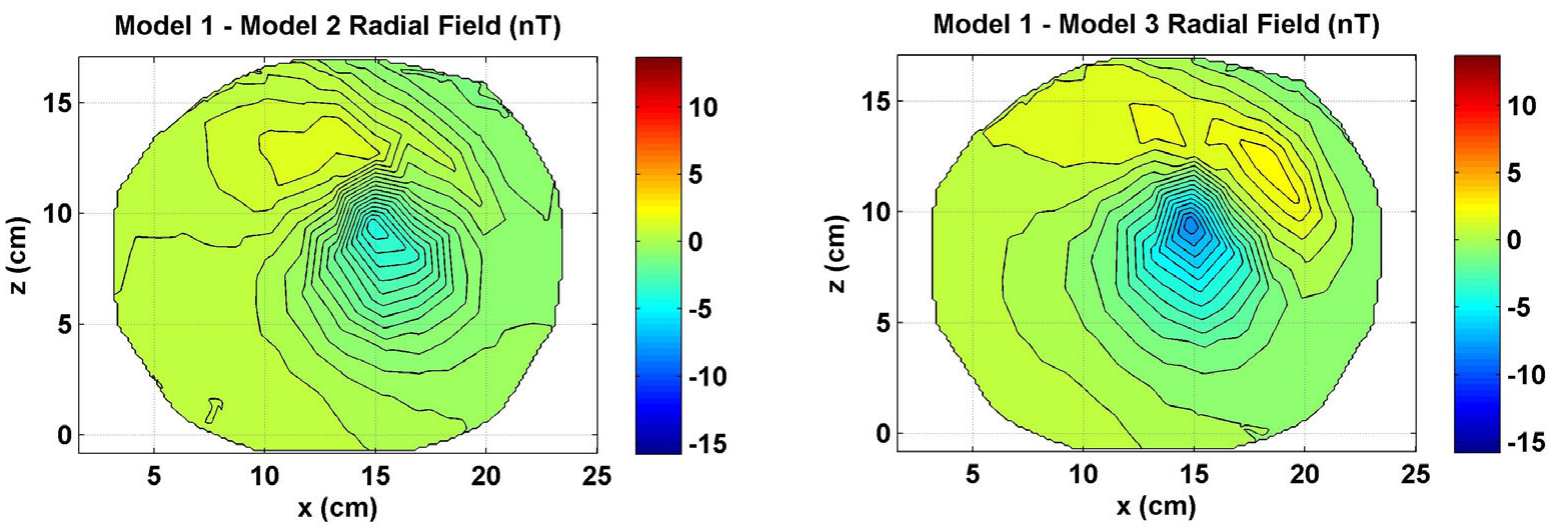
Differences in the radial magnetic fields between the references model and the other two models. Model 1 was used as a reference model. (Left) differences between the Model 1 and Model 2, (right) differences between the Model 1 and Model 3. All values are in nano Tesla (*nT*).

The contour plots of the magnetic fields for a representative dipole of all three models are shown in Figures 2 to 9. These contour plots are for a typical dipole in the motor cortex. This particular dipole for which the contour plots are shown was located at a depth of 3.2 cm from the scalp surface. The magnitude values are in nano Tesla (*nT*) in all of the plots of Figures 2 to 9.

The *x *component of the magnetic field (*B*_*x*_) due to the dipolar source normal to the cortical surface is given in Figure 2. The top left plot is for the magnetic field due to the source current. The magnetic field due to the volume currents in the Model 1 is given in top right plot. Similarly the magnetic fields due to the volume currents for the Model 2 and the Model 3 are shown in the bottom left and right plots, respectively. The location of the positive and negative peaks for the volume currents is diametrically opposite of the source currents magnetic field plot. This is expected because the returning volume currents (extracellular) flow opposite to the direction of the MEG source currents (intracellular). The magnetic field due to the source current has the largest magnitude as compared to the volume currents magnetic fields. Its positive and negative contour peak values are 50 *nT *and -53.2 *nT*. The positive peaks for the models 1 and 3 are almost half in magnitude as compared to positive peak value (53.57 *nT*) of the source current. The negative peak values for the Model 1 and 3 are very close in magnitude to the negative peak of the magnetic fields of the source current. In comparison, the magnitude values for the Model 2 are much smaller as compared to source current magnetic fields. This emphasizes the fact that improper segmentation of the gray and white matter boundaries severely influences the scalp magnetic fields. The magnetic field profiles for the volume currents are slightly different for all three models. These differences are largest for the Model 3(bottom right plot) as compared with the Model 1 (top right plot). The zero-crossing line is almost horizontal for the Model 1 while it slightly deviates from the horizontal position for the Model 2. This deviation is more pronounced for the Model 3. There are slight differences in the location of the positive and negative peaks for all three models.

The total *x *component of the magnetic field, i.e., the sum of the magnetic fields due to the source and the volume currents are given in Figure 3. These fields were also used for inverse source localizations. The magnetic field of the source current does dominate the contour plot of all three models. However, there are noticeable differences in the contour plots of all three models. These are due to the spreading patterns of the volume currents in each model. The positive peak in red color and the bottom negative peak in blue color is due to the source current. The upper negative peak is due to the volume currents. It is weaker than the source current negative peaks in all three contour plots. The magnitude scale is kept same in Figures 3, 5 and 7. This way one could compare the relative magnitudes of *x, y*, and *z *components of magnetic fields. The maximum value is 53.7 nT for the Model 1 in Figure 3 and the minimum value is -71.6 nT for the Model 1 in the Figure 7. Because of this a magnitude scale of -75 *nT *to +75 *nT *was chosen for Figures 3, 5 and 7.

The *y *component of the magnetic field (*B*_*y*_) for the same dipole is given in Figure 4. The *y *axis is pointing downward from the top of the head. The primary dipolar current is normal to the cortical surface. The contour patterns of the volume currents are very different for all three models. This shows that the *y *component of the magnetic is significantly affected by the volume currents in a model. The sum of the source and volume currents magnetic fields is given in Figure 5 for all three models. Once again, the magnetic field of the source currents dominate the contour plots of all three models. Also, all three contour plots are different signifying that the total magnetic field is influenced by the magnetic fields of volume currents.

The *z *component of the magnetic fields (*B*_*z*_) are shown in Figure 6. The contour patterns of the volume currents are very similar for all three models. The peak magnitude values are lower for the Model 2 as compared to the Models 1 and 3. This will imply that the current flow at gray and white matter boundary strongly influences the scalp magnetic fields. Figure 7 shows the total *z *component of the magnetic fields. The contour plots for all three models are very significantly different. In particular, the Model 1 magnetic field in the left plot has very distinctive features as compared to the Model 2 (middle plot) and the Model 3 (right plot) magnetic field plots. This will suggest that a more heterogeneous head model will help in better accounting of the magnetic fields generated due to volume currents.

### Radial magnetic fields

The radial magnetic fields which are usually measured by a whole head SQUID biomagnetometer are given in Figure 8. These include magnetic fields due to the source and volume currents both. All three models exhibit a dipolar magnetic field pattern, but the contour profiles are different. The differences are more discernable for positive contours in red color as compared to the negative contours in blue color. The Model 1 was used as a reference model for a comparative analysis of the inverse source localizations. For this reason, the differences in the radial magnetic fields of Model 2 and Model 3 with respect to the Model 1 are given in Figure 9. The magnitude scale is same in Figures 8 and 9. The magnetic field due to the source currents is same for all three models while the magnetic field due to the volume currents is different for each model. Thus, the contour plots in Figure 9 actually reflect the differences in volume current flow patterns of Model 1 versus Model 2 (left plot) and the Model 1 versus Model 3 (right plot). The differences between the Model 1 and Model 2 are in the range of 2.5 *nT *to -5 *nT*. The patterns of the contours are also different in the left and right plot. This will suggest that the Model 2 and Model 3 will produce different results while doing the inverse source localization.

### Inverse results

**Figure 10:**
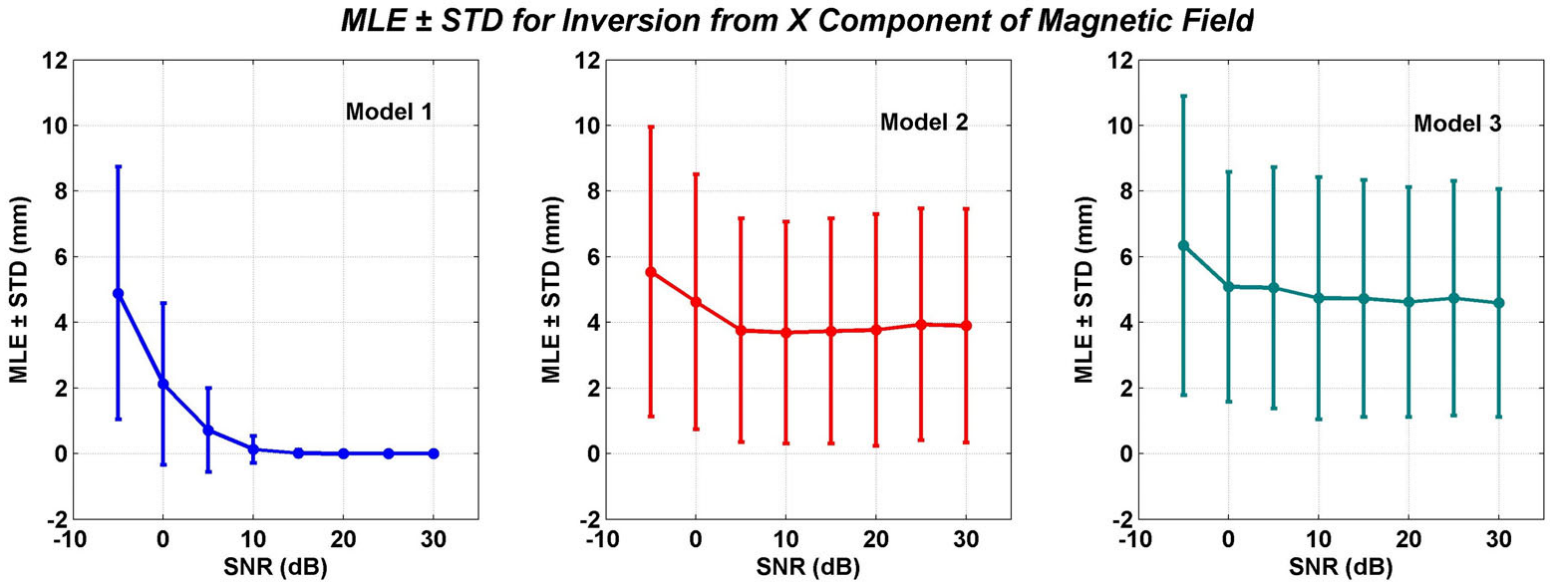
Mean localization errors (MLEs) and standard deviations (STDs)of three models for inversion from the total *x *component of the magnetic field. This included magnetic fields due to the source and volume currents. All values are in millimeters *(mm)*. These MLEs and STDs are averaged over one hundred trials for source localizations within the motor cortex.

**Figure 11:**
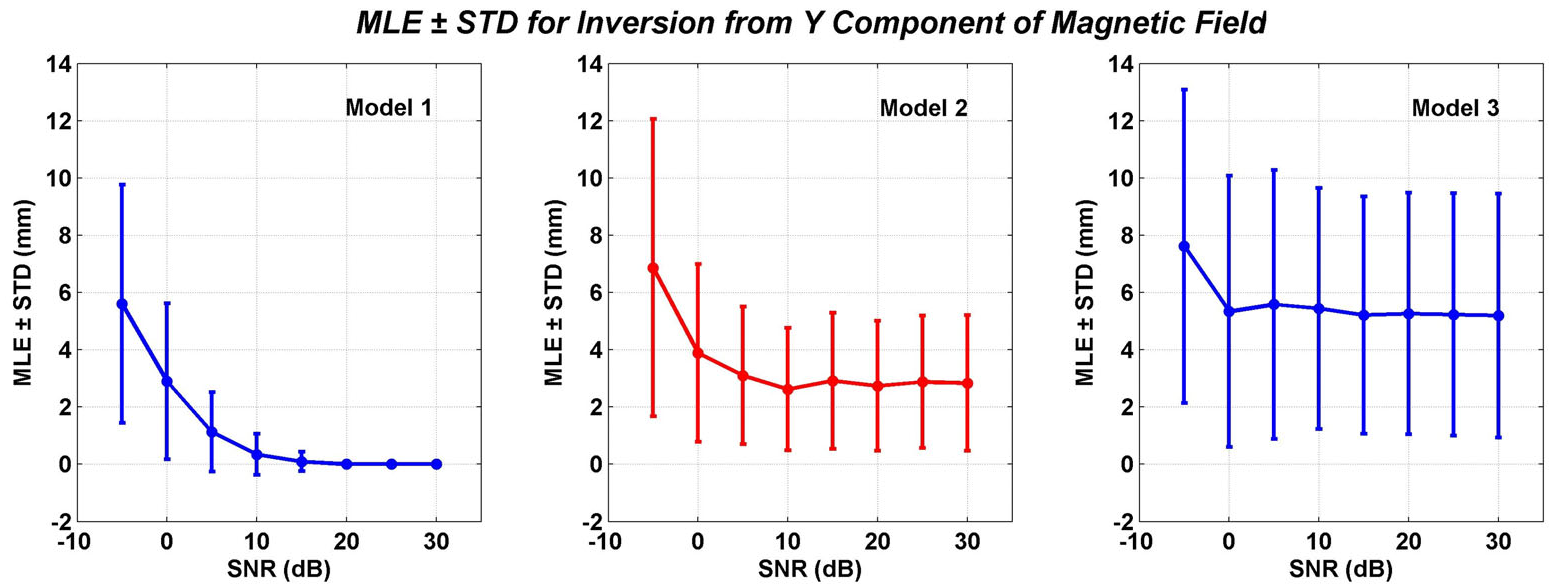
Mean localization errors (MLEs) and standard deviations (STDs) of three models for inversion from the total *y *component of the magnetic field. All values are in mm.

**Figure 12:**
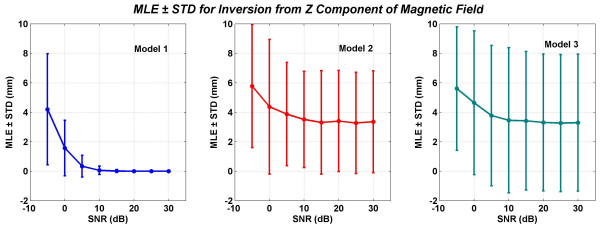
Mean localization errors (MLEs) and standard deviations (STDs) of three models for inversion from the total *z *component of the magnetic field. All values are in mm.

**Figure 13:**
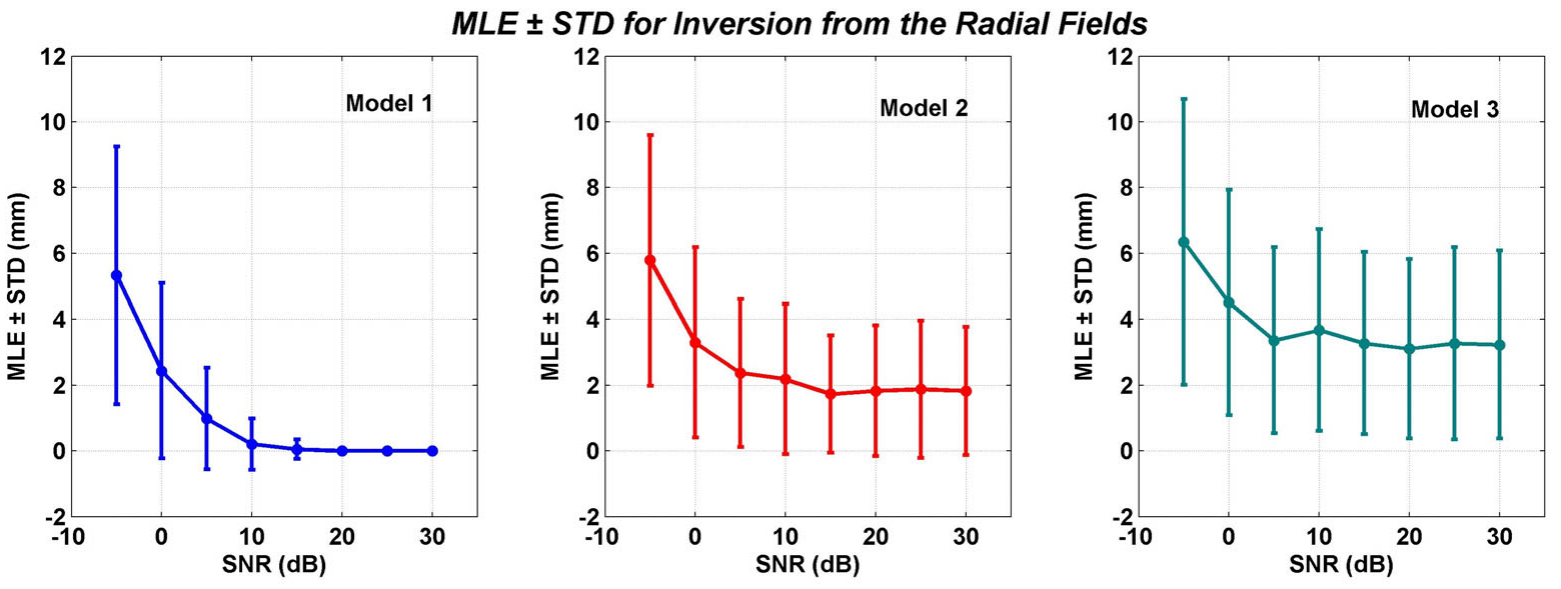
Mean localization errors (MLEs) and standard deviations (STDs) when inverse analysis was performed with the radial component of the magnetic field. All values are in mm.

Mean localization errors (MLEs) and standard deviations (STDs) for source localizations from the magnetic fields are given in figures 10 to 13. These values are averaged over 100 trials of source localizations in a volume of 3 cm cube in the motor cortex. In general, the results shown in Figures 10 to 13 are similar. The Model 1, which is the reference model, performs the best. The Model 2 performs better than the Model 3. The STD values are large for Model 2 and 3 as compared with the Model 1. At few data points for the Model 1, the combined MLE-STD values become negative. One should note that in such situations the minimum MLE will be zero because the MLEs can not be less than zero.

The MLEs and STDs for inversion from *B*_*x *_are given in Figure 10. As expected, the Model 1 performs the best because it was also used for generating the simulated MEGs for inversion. However, the simulated MEG is not the same as the lead field of the Model 1. The simulated MEG data for each trial had a random intensity with an added uncorrelated Gaussian noise in the range of -5 to 30 dB. Thus the simulated MEG data used for inversion is significantly different from the lead fields of the Model 1. Even then, the Model 1 does perform better than the other models. For most of the realistic measurement situations, the SNR values will be in the range of 0 to 10 dB. For a comparative analysis, we will look at the values at 5 dB of SNR. The MLE ± STD at 5 dB of SNR for models 1, 2 and 3 are: 0.71 ± 1.28, 3.75 ± 3.41 and 5.05 ± 3.68, respectively. These values show that Model 2 performs better than Model 3. The standard deviation values are large for the Model 2 and 3 at all levels of SNR.

The MLEs and STDs for inversion from *B*_*y *_are given in Figure 11. Once again the Model 2 performs better than the Model 3. The MLE and STD values are lesser at all SNR levels for the Model 2 as compared with the Model 3. The MLE ± STD at 5 dB of SNR for models 1, 2 and 3 are: 1.12 ± 1.39, 3.1 ± 2.41 and 5.58 ± 4.7, respectively.

The MLEs and STDs for inversion from *B*_*z *_are given in Figure 12. Here the performance of the Model 2 and 3 are very similar. The MLE ± STD at 5 dB of SNR for models 1,2 and 3 are: 0.38 ± 0.74, 3.87 ± 3.5 and 3.77 ± 4.76, respectively.

The MLEs and STDs for inversion from the radial magnetic fields are given in Figure 13. Once again the performance of the Model 2 is better than the Model 3 at all levels of SNR. The MLE ± STD at 5 dB of SNR for models 1, 2 and 3 are: 1.2 ± 1.36, 2.37 ± 2.68 and 3.69 ± 2.67 mm, respectively.

## Discussion

These results suggest that head model complexities influence both the forward MEG simulations and the inverse source localizations. In a comparative analysis, the Model 3 has larger source localization errors as compared to the Model 1 or 2. In Model 2, the difference between the gray and white matter boundary was eliminated. This has significantly changed the forward MEG field patterns and increased the source localization errors. This would imply that proper segmentation of the gray and white matter tissue boundary is needed to reduce source localization errors from MEG data sets.

Our MLE results also show that localization errors increase as the complexity of the model decreases. The fat, muscle and soft bone structures are not included in the Model 3 and this model has larger source localization errors as compared to the Model 1 or Model 2. This suggests that highly heterogeneous finite element models of the head have a potential to better simulate neuromagnetic fields and also could perform better in MEG source localizations. This work was limited to dipoles in the motor cortex area. However, one could expect similar results for dipoles located in other parts of the cortex. This study needs to be extended to other parts of the brain.

The MLEs for the Model 1 are slightly lower for inversion from the Cartesian components as compared with the radial component. Please refer to the Model 1 results, left plot in figures 10,11,12, 13. In contrast, this is different for Models 2 and 3 where the MLEs are less for the radial component as compared with Cartesian components. This could be related to how the cortical volume in the Model 1 modifies the spread of the volume currents which in turn influences the scalp magnetic field profiles. In general, inversion results are better if the field profiles have more spatial features, i.e., more higher spatial frequencies. Comparing the total magnetic fields for the Model 1 in Figures 3, 5, 7 and 8, the Cartesian magnetic field profiles are slightly richer in features as compared to the radial magnetic field profile. However, this could only be true for this particular subject. This needs to be further examined with models constructed from MRI data of several subjects and a statistical analysis should be performed.

These model dependant results on MEG simulations should also be compared with the tissue conductivity related results where one changes the tissue conductivity in steps and examines the changes in the MEGs [7,19-22]. Previous studies have not eliminated tissue boundaries, but they have used incremental changes in the tissue conductivities or have used upper and lower bounds of the tissue conductivity values [20]. Also, detailed contour maps of simulated MEGs are not available in previous studies to perform a comparative analysis. In general, previous studies have found that both the forward and inverse results are severely influenced by changes in the conductivity of skull bone, CSF, gray and white matter. In particular, conductivity of skull bone [20-22] and the skull anisotropy [21] severely influences the EEG and MEG simulations and inverse source reconstructions. Conductivity related inverse localization errors could be of the order of 2.35 mm to 9.59 mm [20]. Our results also show that more complex head models have smaller localization errors. This suggests that highly heterogeneous finite element models of the head are needed to reduce the source localization errors.

In an earlier study[21], we have shown that changes in cerebellum conductivity has a negligible influence on scalp EEG or MEG. Only tissues between the source and the sensor locations, such as, scalp, fat, skull and muscle severely influence the MEGs and EEGs[1]. Thus, replacing the cerebellum with CSF, gray matter or white matter will have negligible influence on our results reported here for MEG simulations as well as in our previous paper [1] on EEG simulations. For model development purposes, one could replace cerebellum with CSF, gray or white matter and it will have negligible influence for the sensors located on the top covering most of the head above eyes and ears. However, changes in cerebellum conductivity has a possibility to influences the EEG or MEG sensors located on the back of the neck.

The tissue conductivity values used in our forward simulations are based on the averaged values available in the literature [11-13]. The *in-vitro *skull conductivity has been measured again recently [23] and was found to be 0.015 S/m, or, equivalently, 15E-5 S/cm. In our modeling work we are using hard and soft skull bone resistivities of 16,000 and 2,180 Ohm cm, respectively. In a rough approximation, assuming equal volumes of the hard and soft skull bone, the average skull conductivity will be 9,090 Ohm cm. This is equivalent to 11E-5 S/cm which is very close to the recently measured value of 15E-5 S/cm [23]. Based on a three compartment, brain, skull and head, boundary element model they [23] also estimated the tissue conductivity ratios. It was found that the conductivities of the brain, the skull and the scalp had a ratio of 1 : 1/15 : 1. Similarly, using a 3-shell spherical model [24] the brain/skull conductivity ratio was estimated to be 1/(25 ± 7). In our models, the average conductivity of combined brain gray and white matter will be 2E-3 S/cm. This will give us a brain/skull conductivity ratio of 1/18 which is in between the ratios suggested by these authors [23,24]. One also needs to note that these conductivity ratios have been estimated with a 3-shell spherical or a three compartment boundary element method model. Estimation of brain/skull conductivity ratio or tissue conductivities with highly heterogenous models has not been performed so far and, if done, could come out to be very different from the previously reported values. The anisotropic conductivities of gray and white matter also influence the EEG and MEG simulations [9,25]. It needs to be examined how the model complexity combined with the tissue anisotropies influence the forward and inverse MEG simulations.

In general, the MEG data has a SNR in the range of 0 to 10 dB. As stated earlier, for the radial magnetic fields (fig. 13) the MLE ± STD at 5 dB of SNR for models 1, 2 and 3 are: 1.2 ± 1.36, 2.37 ± 2.68 and 3.69 ± 2.67 mm, respectively. This should be compared with the coregistration errors of the MEG sensor locations within the MR images. These coregistration errors are approximately 2 mm [26]. The Model 2 and 3 localization errors are larger than 2 mm while that of the Model 1 is less than the 2 mm. This will imply that by use of a better model one can bring down the localization errors very close to the limit of coregistration errors.
